# Development of a Mediterranean diet score adapted to Japan and its relation to obesity risk

**DOI:** 10.3402/fnr.v60.32172

**Published:** 2016-11-01

**Authors:** Masao Kanauchi, Kimiko Kanauchi

**Affiliations:** 1 Department of Health and Nutrition, Faculty of Health Science, Kio University, Koryo-cho, Japan; 2 Department of Internal Medicine, Narahigashi Hospital, Tenri, Japan

**Keywords:** Mediterranean diet, Japanese, diet quality, obesity

## Abstract

**Background:**

The Mediterranean diet (MD) is well known as a healthy diet that protects against several chronic diseases. However, there is no appropriate and easy index to assess adherence to the MD pattern in Japan.

**Objective:**

The aim of this study was to develop a novel instrument to measure MD adherence adapted to a Japanese diet and to examine its association with overweight/obesity risk.

**Methods:**

A cross-sectional nutritional survey provided the data for construction of a novel MD score. In total, 1,048 subjects who were employees and university students, aged 18–68 years (645 men and 403 women), completed a 58-item brief-type self-administered dietary history questionnaire. We constructed a Japanese-adapted MD score (jMD score) focusing on 13 components. Adherence to the jMD was categorized as low (score 0–4), moderate (5–7), or high (8–13).

**Results:**

Men had higher jMD scores than women, and adherence to the jMD score increased with age. Only 11.6% of subjects showed high adherence to the jMD, whereas 29.6% showed low adherence. A higher jMD adherence was associated with a higher intake of favorable nutrients with the exception of salt. The jMD adherence was significantly associated with a reduced likelihood of having overweight/obesity for the highest category compared with lowest category (odds ratio [OR] 0.50, 95% confidence interval [CI] 0.30–0.85, *p-trend*=0.017) after adjusting for age, sex, smoking, physical activity, alcohol intake, and hypertension. A two-point increment in jMD score was related to a reduced likelihood of having overweight/obesity with an odds ratio of 0.76 (95% CI 0.65–0.90, *p*=0.002).

**Conclusions:**

Our novel jMD score confirmed reasonable associations with nutrient intakes, and higher MD adherence was associated with a lower prevalence of overweight/obesity.

In the last few decades, the Mediterranean diet (MD) has been recognized as a healthy diet that protects against several chronic diseases (1–4). The traditional MD is characterized by a high consumption of cereals (mostly whole grains), vegetables, legumes, fruits and nuts, and olive oil; a moderate intake of fish; a low-to-moderate intake of dairy products; a low consumption of red and processed meat; and a moderate alcohol intake from red wine (5, 6). Although adherence to the MD has been studied in relation to a lower rate of all-cause mortality and a reduced risk of cancer, metabolic diseases, and cardiovascular disease (7, 8), little is known about the efficacy in a non-Mediterranean population.

The vegetable dietary pattern in Asian populations may also reduce cardiovascular risks (9, 10). A traditional Japanese diet, characterized by whole grains, colorful vegetables, soy products (e.g. tofu), fish, and konbu seaweed, has also been recognized as healthy (11, 12). Some components of this dietary pattern are similar to that of the MD. To date, the prevalence of obesity is increasing rapidly in Japan as well as in Western countries. Over the past few decades, the change from a traditional diet to a westernized dietary pattern had led to an increase in the rate of diabetes and cardiovascular disease in Japan (13). Therefore, switching from a modern dietary pattern to a healthy MD-like pattern may play an important role in the reduction of obesity (14).

Various indexes to assess the adherence to the MD have been proposed and used in nutritional epidemiologic studies (15, 16). However, there are cultural food differences between Mediterranean/European countries and Japan, making these indexes less applicable for Japan. A valid and easy assessment tool should be constructed that can capture the MD pattern in Japan. The aim of this study was to develop a novel instrument to measure MD adherence and compare it to previously reported MD scores. We also examined the relationship of scores on this instrument with overweight/obesity risk.

## Methods

### Subjects

In total, 1,002 employees at eight workplace settings and 62 university students were invited to participate in this study. Most employees were industrial workers, office workers, formal caregivers, or nursing staff. Of these, we excluded one subject who was older than 70 years, two subjects who did not complete the diet history questionnaire, 11 subjects who had implausibly low or high estimated caloric intake (<700 or >4,500 kcal per day for men and <600 or >3,500 kcal per day for women), and two subjects who had missing information for factors needed for statistical adjustment. Finally, 1,048 participants aged 18–68 years (645 men and 403 women) were included in this analysis. Study protocols were approved by the Institutional Review Board of Kio University, and written informed consent was obtained from each participant.

### Dietary assessment

Habitual food consumption and nutrient intake were assessed using the brief-type self-administered diet history questionnaire (BDHQ) (17, 18). The BDHQ is a four-page fixed-portion questionnaire, and food and beverage items contained in the BDHQ were selected from foods commonly consumed in Japan, mainly from a food list used in the National Health and Nutritional Survey of Japan (19). The questionnaire asks about the consumption frequency of 58 food and beverage items and requires that participants recall their dietary habits over the previous 1-month period. Participants were asked to choose from seven possible answers to indicate how often they had consumed specific foods during the past month (never, <one time per week, once per week, two to three times per week, four to six times per week, once per day, and more than twice per day). Combined with standard serving sizes, the intake frequencies were converted into the average daily intake for each food item. Values for the intake of nutrients and energy were estimated based on the food items asked about on the questionnaire and the corresponding food composition list in the Standard Tables of Food Composition in Japan (20).

### Mediterranean diet score

We developed a Japanese-adapted MD score (jMD score) by adapting the updated MD pyramid (21) to Japanese eating behaviors; we selected only some components of the MD pyramid and modified some cutoff values to more accurately reflect the standard Japanese diet. This score is based on 13 components: grains (including rice, bread, Japanese wheat noodles, buckwheat noodles, spaghetti, macaroni); vegetables (e.g. green-yellow vegetables, leafy vegetables, root vegetables, cruciferous vegetables, vegetable pickles); fruits (citrus, persimmons, strawberries, kiwifruit, other fruit); legumes (tofu, fermented soybeans, miso); fish (oily fish, non-oily fish, canned tuna, dried fish, fish paste products, shellfish); dairy products (milk, low-fat milk, yogurt); potatoes (all types); eggs; poultry; red meat and processed meat (beef, pork, ham, sausage, bacon); sweets (Japanese confectionaries, Western-type confectionaries, ice creams); alcohol (beer, sake, shochu, wine, whiskey); and the ratio of monounsaturated fatty acids to saturated fatty acids. One point was given to subjects who met criteria for 1) consumption of three to seven servings of grains per day, 2) consumption of five or more servings of vegetables per day, 3) consumption of two or more servings of fruits per day, 4) consumption of 30 g or more of legumes per day, 5) consumption of 84 g or more of fish per day for men and 66 g or more of fish per day for women, 6) consumption of 1.5–2.5 servings of dairy products per day, 7) consumption of potatoes one to three times per week, 8) consumption of poultry two to three times per week, 9) consumption of eggs two to three times per week, 10) consumption of meat or meat products less than three times per week, 11) consumption of sweets less than two times per week, 12) consumption of 10–30 g of alcohol per day for men and 5–15 g of alcohol per day for women, and 13) a ratio of 1.5 or more of monounsaturated fatty acids to saturated fatty acids. Otherwise, a score of zero was assigned. This resulted in scores ranging from 0 to 13, with a higher score indicating better adherence to the jMD. Adherence to the jMD was categorized as low (score 0–4), moderate (score 5–7), or high (score 8–13).

To compare with previously reported MD scores, three MD scores were calculated: the Mediterranean diet scale (T-MDS) proposed by Trichopoulou et al. (22), the Mediterranean Dietary Quality Index (MedDQI) proposed by Gerber (23), and the Mediterranean diet score (S-MDS) proposed by Sofi et al. (24).

### Other variables

Body mass index (BMI) was calculated as weight in kilograms divided by the square of height in meters. Overweight/obesity was defined as BMI ≥25 kg/m^2^, because the percentage of persons with BMI ≥30 kg/m^2^ is rare in Japan (25). A self-reported questionnaire was used to assess current smoking status (yes, no), physical activity (active, sedentary), and hypertension (yes, no). Alcohol consumption (frequency and daily consumption) was evaluated by BDHQ information and was categorized as low (men, <10 g per day; women, <5 g per day), moderate (men, 10–30 g per day; women, 5–15 g per day), or high (men, >30 g per day; women, >15 g per day).

### Statistical analysis

Data are presented as means±SD or as percentages. Differences between two groups were compared using the Student's *t*-test for continuous variables or the chi-squared test for categorical variables. Differences of food or nutritional parameters across the MD adherence categories were assessed by one-way analysis of variance. Logistic regression analysis was used to evaluate the risk of having obesity. Linear trends were calculated using the median score of the categories of MD adherence. *P*-values <0.05 were considered statistically significant. All statistical analyses were performed using the SPSS statistical version 21.0 (IBM Corp., Armonk, NY).

## Results

The mean age of subjects was 42.6±11.4 years (range, 18–68 years), and the prevalence of overweight/obesity (BMI ≥25.0 kg/m^2^) was 27.8%. The mean jMD score was 5.45±1.77 (range, 0–11), and scores were symmetrical and normally distributed (data not shown). Men had higher jMD scores than women, and a greater adherence to the jMD was shown in older subjects than in younger subjects (Table 1).

**Table 1:** Association of personal characteristics of study subjects with mean Mediterranean diet score adapted to Japan (jMD)

	Number	jMD score	*p*
Sex			<0.001
Men	645	5.66±1.83	
Women	403	5.11±1.62	
Age group (years)			<0.001
18–29	151	4.92±1.66	
30–39	215	5.42±1.71	
40–49	392	5.38±1.81	
50–59	241	5.80±1.76	
60 +	49	6.00±1.58	
Overweight/obesity^a^			0.150
Yes	291	5.32±1.78	
No	757	5.50±1.76	
Smoking status^b^			0.398
Yes	252	5.54±1.81	
No	732	5.43±1.75	
Physically active^b^			0.314
Yes	445	5.40±1.77	
No	539	5.51±1.77	
Alcohol intake^c^			0.823
No or low	670	5.24±1.69	
Moderate	148	6.63±1.83	
High	230	5.27±1.65	
Hypertension			0.052
Yes	241	5.65±1.90	
No	807	5.39±1.72	

Data are means±SD.

a Overweight/obesity was defined as BMI ≥25 kg/m^2^.

b Data on smoking status and physical activity status were missing for 64 subjects.

c Alcohol consumption was categorized as low (men, <10 g per day; women, <5 g per day), moderate (men, 10–30 g per day; women, 5–15 g per day), or high (men, >30 g per day; women, >15 g per day).

When subjects were classified by jMD adherence categories, only 11.6% of subjects showed high adherence to the jMD, whereas 29.6% showed low adherence. A higher jMD adherence was associated with a higher intake of protein, polyunsaturated fatty acids, dietary fiber, salt, potassium, calcium, magnesium, iron, vitamin D, folate, and vitamin C and a lower intake of saturated fats and sucrose (Table 2).

**Table 2:** Food and nutrient intake by the adherence levels to the Mediterranean diet adapted to Japan (jMD)

Food/nutrients	jMD score 0–4 (*n*=310)	jMD score 5–7 (*n*=616)	jMD score 8–13 (*n*=122)	*p*
Grains (g/day)	552±235	548±176	570±145	0.515
Potatoes (g/day)	40±40	55±41	71±44	<0.001
Legumes (g/day)	42±37	65±42	84±56	<0.001
Vegetables (g/day)	214±123	303±139	365±158	<0.001
Fruits (g/day)	92±93	119±125	158±141	<0.001
Fish (g/day)	67±43	94±47	127±63	<0.001
Meat (g/day)	89±54	95±43	95±37	0.194
Dairy (g/day)	166±154	143±119	132±124	0.013
Eggs (g/day)	48±31	51±32	46±27	0.179
Energy (kcal/day)	2,318±305	2,366±296	2,456±247	<0.001
Protein (%E)	13.3±2.8	14.5±2.5	15.6±2.5	<0.001
Fat (%E)	25.7±6.7	25.8±5.4	26.2±4.4	0.780
Carbohydrate (%E)	55.3±8.9	53.7±7.6	53.0±6.0	0.006
SFA (%E)	7.4±2.4	6.7±1.8	6.3±1.4	<0.001
MUFA (%E)	9.3±2.6	9.4±2.2	9.5±1.8	0.740
PUFA (%E)	5.8±1.4	6.4±1.3	6.9±1.3	<0.001
M/S ratio	1.30±0.23	1.42±0.24	1.55±0.25	<0.001
Dietary fiber (g/day)	11.3±3.6	13.9±4.1	16.2±4.2	<0.001
Sucrose (g/day)	20.6±12.2	17.7±10.4	15.6±10.9	<0.001
Salt (g/day)	12.4±3.2	13.7±3.2	15.9±3.9	<0.001
Potassium (mg/day)	2,509±732	2,997±805	3,502±824	<0.001
Calcium (mg/day)	545±230	608±197	689±215	<0.001
Magnesium (mg/day)	259±61	303±62	353±75	<0.001
Iron (mg/day)	7.9±2.1	9.3 ±2.2	10.5±2.3	<0.001
Vitamin D (µg/day)	11.5±8.0	15.3±7.9	20.3±9.8	<0.001
Folate (µg/day)	327±119	402±133	472±140	<0.001
Vitamin C (mg/day)	100±48	130±58	159±63	<0.001

Data are means±SD.

SFA, saturated fatty acids; MUFA, monounsaturated fatty acids; PUFA, polyunsaturated fatty acids; S, saturated; M, monounsaturated.

With respect to the correlations among the four MD indexes, the highest correlations were observed between the jMD score and T-MDS, followed by the T-MDS and S-MDS, and the T-MDS and MedDQI. Lower correlations were seen between the jMD score and MedDQI, and the jMD score and S-MDS (Table 3).

**Table 3:** Correlation among indexes of adherence to the Mediterranean diet

	jMD score (correlation *p* coefficient)	T-MDS (correlation *p* coefficient)	MedDQI (correlation *p* coefficient)	S-MDS (correlation *p* coefficient)
jMD score	1	–	–	–
T-MDS	0.490 (<0.001)	1	–	–
MedDQI	0.287 (<0.001)	0.457 (<0.001)	1	–
S-MDS	0.184 (<0.001)	0.460 (<0.001)	0.405 (<0.001)	1

jMD, Mediterranean diet score adapted to Japan; T-MDS, Mediterranean diet scale proposed by Trichopoulou et al. (22); MedDQI, Mediterranean Dietary Quality Index proposed by Gerber (23); S-MDS, Mediterranean diet score proposed by Sofi et al. (24).

The jMD adherence was significantly associated with a reduced likelihood of having overweight/obesity for the highest category compared with lowest category (odds ratio [OR] 0.50, 95% confidence interval [CI] 0.30–0.85, *p-trend=*0.017) after adjusting for age, sex, smoking, physical activity, alcohol intake, and hypertension (Table 4). A two-point increment in jMD score was related to a reduced likelihood for having overweight/obesity with an odds ratio of 0.76 (95% CI 0.65–0.90, *p*=0.002).

**Table 4:** Association of jMD score with the likelihood (odds ratios and 95% CIs) of having overweight/obesity

	Total, *n* (%)	Overweight/obesity, *n* (%)	Crude	Model 1: OR (95% CI)	Model 2: OR (95% CI)	Model 3: OR (95% CI)
jMDS score adherence						
Low (score 0–4)	310 (29.6)	87 (28.1)	1	1	1	1
Moderate (score 5–7)	616 (58.8)	175 (28.4)	1.02 (0.75–1.38)	0.96 (0.71–1.16)	0.89 (0.65–1.22)	0.87 (0.63–1.20)
High (score 8–13)	122 (11.6)	29 (23.8)	0.80 (0.49–1.30)	0.71 (0.43–1.16)	0.56 (0.34–0.92)	0.50 (0.30–0.85)
*p-trend*			*0.504*	*0.236*	*0.037*	*0.017*

Data are odds ratio (95% CI).

Model 1 is adjusted for age. Model 2 is adjusted for age and sex. Model 3 is adjusted for age, sex, smoking status, physical activity, alcohol intake, and hypertension (yes/no).

jMD, Mediterranean diet score adapted to Japan.

## Discussion

This is the first study to develop a novel 13-item MD score adapted to Japan dietary habits and to investigate its association with overweight/obesity. The impact of poor dietary habits on health is an important focus of contemporary health promotion strategies. The MD is now widely promoted for the prevention of cardiometabolic diseases (1–4). Adherence to the MD has been shown to be inversely associated with all-cause mortality, cardiovascular disease mortality, and cancer incidence (7, 8), but evidence of MD adherence in a non-Mediterranean population, especially East Asians, is limited (26–28). A study conducted in Korean adults (26) reported that adherence to the MD evaluated using an alternative MD score was related to lower oxidative stress biomarkers. A study conducted in Chinese elderly (27) reported that adherence to the MD was associated with a lower risk for hip fractures. A study conducted in Hong Kong (28) reported that no association was observed between the MD score and 4-year incident frailty in community-dwelling older people. However, adherence to the MD in Japanese people has not been precisely explored. One of the reasons is thought to be the lack of an appropriate assessment tool for this population. In the Mediterranean and European countries, several indexes for assessing MD adherence have been reported (7). The T-MDS, created by Trichopoulou et al. in 1995 (5) and updated thereafter (6, 22), is widely known and is based on consumption of nine items. Other examples are the MedDQI (23), the S-MDS (24), the alternate MD score (29), and the MedDietScore (30) that include 7 components, 9 components, 9 components, and 11 components, respectively. It is interesting to note that the components of these scores and their scoring systems differ across studies (5, 6, 22–24, 29, 30). In addition, there is no consensus in the definition of the MD as represented by country-specific features (31).

We created the jMD score based on the updated MD pyramid (21). We incorporated white meat (in Japan, this consists mainly of poultry), potatoes, eggs, and sweets into our score, because the updated MD pyramid recommends that the first three foods should be limited to weekly consumption, and sweets should be consumed occasionally in small amounts. Conversely, we did not incorporate olive oil and nuts into our score, because the BDHQ used in the present study could not calculate the amount of these foods. In addition, consumption of olive oil and nuts are low in Japan. We alternately used an indirect parameter of the monounsaturated fatty acids to saturated fatty acids ratio in the present jMD score, which has been used in several previous studies (5, 6, 22, 29).

When creating the jMD score, we modified some cutoff values to consider Japanese eating habits. First, the MD pyramid today represents a weekly consumption of two or more servings of fish and shellfish (21). However, in general, Japanese people eat a lot of fish and shellfish (32). Almost all of participants might exceed this cutoff level of fish intake. We amended the cutoffs for fish intake with an upward adjustment based on the mean consumption levels of the Japanese population (≥84 g per day for men and ≥66 g per day for women) (33). Second, most of the previously reported MD scores consider alcohol intake, especially wine. Japanese people commonly consume several kinds of alcoholic beverages such as sake (Japanese rice wine), beer, or shochu (Japanese distilled alcohol beverage), but the proportion of wine drinkers is relatively low. For this reason, we assessed moderate consumption of all amount of alcohol beverages.

One of the strengths in the present study is that we chose predefined absolute cutoffs of consumption of each food to estimate jMD adherence. In most previous reported MD scores (5, 6, 22, 26–29), the cutoff points are based on the sex-specific medians among the sample population. Under these considerations, their scores are highly dependent on the sample characteristics. Because of the heterogeneity of their cutoff points in each MD score, a result of one study could not be comparable with another study performed in a different cohort even in the same country. This could result in misleading data regarding the association between MD adherence and health outcomes. Indeed, our jMD scores were fairly correlated to T-MDS, but remaining correlations were poor. This is in line with a previous report (16) that compared data from 10 different MD scores in which only three correlations were high but the remaining correlations were fair or poor. This finding implies that the previous proposed MD scores are widely different. As mentioned above, the cutoff point used in the T-MDS reflects their specific sample population, so it is difficult to use in Japanese people, especially in a clinical setting. In contrary, it is noteworthy that our jMD score assigns a value of 0 or 1 to each item according to a predefined absolute cutoff. It seems to be a simple scoring method and to have an advantage of being feasible for assessing or comparing different cohorts studied. Second, we demonstrated an association between jMD adherence and a lower prevalence of overweight/obesity. Two-point increments of jMD score were related to a 24% reduction of having overweight/obesity. Our results indicate that the jMD may help promote healthier eating habits in Japanese people.

On the other hand, we found some unintended results in the present study. The jMD score was shown to be lower among women compared with men, as well as in younger compared with older people. A possible explanation may be that younger women tend to have increased exposure to unhealthy eating habits or unbalanced diets, such as low intakes of grains, legumes, and fish or excess intakes of sweets, resulting in lower jMD scores. In the present study, higher salt intake was observed in the highest jMD category. It is attributable to habitual consumption of higher salt Japanese foods, such as dried fish, canned tuna, and fish paste products (34). In a recent epidemiologic study (35), it is also reported that Japanese adults with higher urinary sodium excretion consumed a significantly greater amount of salty Japanese foods, such as salted fish, miso soup, and Japanese pickles. These salty foods are included in the jMD scores as the fish component, legume component, and vegetable component, respectively. Surprisingly, the MD represents a moderate-to-high salt diet because many of the MD foods such as cheese, bread, some of pastries, anchovies, and salted nuts are pretty salty (36, 37). These foods are recommended to be consumed on a daily basis according to the MD scheme. Nevertheless, the inverse relation of MD adherence and hypertension risk has been shown in epidemiologic studies (38), despite the hidden salt intake supplied from the MD. It may be possible, though not conclusive, that adherence to the MD could be influenced by the impact of salt intake. In this context, based on the updated MD pyramid recommendation (21), use of spices, herbs, and garlic is a good way to contribute to reducing the addition of salt to food. Yet, in Japan, these spices are rarely used despite a frequent use of soy sauce.

This study has several limitations. First, as a cross-sectional study, the present analysis cannot prove a causal relationship between the jMD adherence and the prevalence of overweight/obesity. Second, we assessed the combined risk of overweight and obesity in this study. One reason that we chose the criteria of BMI ≥25 kg/m^2^ is that the percentage of persons with BMI ≥30 kg/m^2^ is rare in Japan. In the National Health and Nutrition Survey in Japan, the percentage of persons with BMI ≥30 kg/m^2^ was only 4.0% in men and 3.3% in women (25); thus, we did not analyze separately the obesity risk of BMI ≥30 kg/m^2^. Third, evaluation of salt intakes was conducted by dietary assessment questionnaires. It can be measured directly with 24-h urine collection, but this is impractical. Our data were estimated from the BDHQ questionnaire and corresponding food composition list in the Standard Tables of Food Composition in Japan (20). In addition, the questionnaire includes a useful information source that asks about the usual cooking methods for fish, meat, and vegetables, preferable use of soy sauce as a seasoning, and consumption of miso soup or soup for noodles. The validity of this BDHQ questionnaire was reported for several nutrient intakes (17, 18). Fourth, MD cuisine is characterized by pasta tossed with vegetables, herbs, and olive oil, dressing salads with olive oil or vinegar, vegetable soups with added olive oil, and chopped or stewed tomatoes and other carotenoid vegetables. Cooking processes might strongly influence antioxidant or anti-inflammatory compounds, including the MD foods (39). For example, fish that is pan-fried in virgin olive oil is frequently eaten in Mediterranean countries, whereas raw fish or grilled fish is preferred in Japan. However, the present study did not take into consideration the difference in cooking procedures between Mediterranean countries and Japan. Fifth, validation analysis between jMD score and plasma biomarkers was not determined because we did not measure plasma biomarkers such as lipids or vitamin levels. Finally, this study was conducted only among adult employees and university students. Although the geographic characteristics of our study sample are similar to the general Japanese population living in an urban or suburban area setting, we did not include subjects in rural communities, such as farm workers or fisherman. Further investigations including subjects with a wider range of occupations is needed.

In conclusion, the present study showed a novel MD score adapted to Japan by confirming reasonable associations with nutrient intakes. The jMD score is also an easy and useful tool to assess jMD adherence. In addition, jMD adherence was associated with a lower prevalence of overweight/obesity. The MD has been recognized as a healthy diet worldwide. It may be useful to encourage the Japanese population to adapt the jMD to prevent chronic diseases. The use of this score in clinical practice or research may contribute to the promotion of a healthier lifestyle.
